# Use of a semi-field system to evaluate the efficacy of topical repellents under user conditions provides a disease exposure free technique comparable with field data

**DOI:** 10.1186/1475-2875-13-159

**Published:** 2014-04-26

**Authors:** Onyango Sangoro, Dickson Lweitojera, Emmanuel Simfukwe, Hassan Ngonyani, Edgar Mbeyela, Daniel Lugiko, Japhet Kihonda, Marta Maia, Sarah Moore

**Affiliations:** 1 Ifakara Health Institute, Box 74, Bagamoyo, Tanzania; 2 Disease Control Department, London School of Hygiene and Tropical Medicine, Keppel Street, London WC1E 7HT, UK; 3 Department of Health Interventions, Swiss Tropical and Public Health Institute, Socinstrasse, 57, CH-4002 Basel, Switzerland; 4 University of Basel, Petersplatz 1, 4003 Basel, Switzerland

**Keywords:** Repellent, *Anopheles arabiensis*, Semi-field system, Efficacy, *N, N*-diethyl-3-methylbenzamide (DEET)

## Abstract

**Background:**

Before topical repellents can be employed as interventions against arthropod bites, their efficacy must be established. Currently, laboratory or field tests, using human volunteers, are the main methods used for assessing the efficacy of topical repellents. However, laboratory tests are not representative of real life conditions under which repellents are used and field-testing potentially exposes human volunteers to disease. There is, therefore, a need to develop methods to test efficacy of repellents under real life conditions while minimizing volunteer exposure to disease.

**Methods:**

A lotion-based, 15% *N, N-*Diethyl-3-methylbenzamide (DEET) repellent and 15% DEET in ethanol were compared to a placebo lotion in a 200 sq m (10 m × 20 m) semi-field system (SFS) against laboratory-reared *Anopheles arabiensis* mosquitoes and in full field settings against wild malaria vectors and nuisance-biting mosquitoes. The average percentage protection against biting mosquitoes over four hours in the SFS and field setting was determined. A Poisson regression model was then used to determine relative risk of being bitten when wearing either of these repellents compared to the placebo.

**Results:**

Average percentage protection of the lotion-based 15% DEET repellent after four hours of mosquito collection was 82.13% (95% CI 75.94-88.82) in the semi-field experiments and 85.10% (95% CI 78.97-91.70) in the field experiments. Average percentage protection of 15% DEET in ethanol after four hours was 71.29% (CI 61.77-82.28) in the semi-field system and 88.24% (84.45-92.20) in the field.

**Conclusions:**

Semi-field evaluation results were comparable to full-field evaluations, indicating that such systems could be satisfactorily used in measuring efficacy of topically applied mosquito repellents, thereby avoiding risks of exposure to mosquito-borne pathogens, associated with field testing.

## Background

Evaluations of topical repellent efficacy against blood feeding arthropods require standardized laboratory and field tests [1-3]. However, conditions in the laboratories are not representative of real life settings where repellents are used. Therefore, experiments carried out in the laboratory may not accurately estimate the efficacy of repellents in the field [4]. Environmental factors such as temperature, humidity and wind speed, all of which affect the effectiveness of repellents, are controlled in the laboratory, but in the field these factors may fluctuate and affect repellent efficacy [5]. As a result, tests carried out in the laboratory ideally should be verified using representative field tests. On the other hand, field evaluations, albeit representative of conditions under which repellents are normally used, can expose volunteers participating in these experiments to mosquito-borne pathogens [6]. Therefore, there is a need to develop methods to test efficacy of repellents under representative user conditions while minimizing volunteer exposure to vector-borne diseases.

There are several techniques that have been proposed for testing topical repellents while reducing human exposure to mosquito bites. These options include: 1) use of synthetic mosquito attractants that mimic human volunteers [7]; 2) use of animals instead of human volunteers [8,9]; 3) use of *in vitro* blood feeding membrane [10-12]; 4) In vitro olfactometry [13]; and, 5) use of a semi-field system (SFS) [14,15]. Although techniques 1 to 4 are convenient because of their high throughput in screening of repellents and do not use human participants, they have well-documented limitations: as the skin is the site of action of topical repellents, and mosquitoes are attracted to cues produced by the host, different hosts will elicit varying degree of responses in the mosquito which will affect both duration and degree of repellency observed [8,10]. The use of *in vitro* blood-feeding membrane is unlikely to give similar results to repellents applied to human skin, as the feeding membrane used in these tests are structurally and physiologically different from the human skin and produce no odour [10]. Use of *in vitro* olfactometry, used mainly to test spatial repellents, is more suitable for screening purposes as it’s used in confined spaces and shorter distances in the laboratory and results cannot be correlated to the field, where there are wide open spaces for the mosquitoes to forage [13]. The use of synthetic blends to test repellency has also proved unreliable as different repellent-blend combinations produced disparate results [7]. Use of SFS may overcome these shortcomings because efficacy tests can be performed in a large enclosure under ambient conditions, allowing mosquitoes to elicit similar behavioural responses as under field conditions. The other advantage of SFS is that it uses mosquitoes reared under laboratory conditions and therefore does not expose volunteers to potential mosquito-borne disease. The species, numbers and physiological status of mosquitoes used in the SFS are standardized to provide more controlled conditions and therefore reduce data variability associated with field studies. However, the effectiveness of SFS has not been evaluated against full-field conditions when testing topical repellents. This study examined whether tests carried out in a SFS would yield comparable results to tests conducted in field setting.

## Methods

### Study area

Semi-field evaluation of repellents was carried out at Ifakara Health Institute (IHI), Morogoro, Tanzania. The field evaluation of repellents was conducted in Mbingu village, Ulanga district, situated 55 km west of Ifakara town at 8.195°S and 36.259°E. Rapid diagnostic test (RDT) results from passive case detection at a local clinic between December 2012 and July 2013 confirmed malaria incidence estimates from the village were 0.67 cases/person-years, (Jabari Mohammed Namamba, pers comm), only one-and-half years after the end of a national campaign to achieve universal coverage with long-lasting, insecticide-treated bed nets (LLINs) [16]. There is high malaria transmission all year round, with peak transmission occurring in the months of May and June after the long rains. The village experiences an annual rainfall of approximately 1,200-1,800 mm and an annual temperature range of between 20 and 32.6°C. The village borders an extensive field cleared for irrigation, which provides an ideal breeding site for malaria vectors [17].

### Semi-field evaluations of topically applied repellents

The semi-field evaluation was carried out in the IHI SFS. A SFS is an enclosed environment, situated in the natural ecosystem of a target vector and exposed to ambient conditions necessary for the completion of the life cycle of the vector. It is made up of a greenhouse frame with walls of mosquito netting and a polyethylene roof, mounted on a raised concrete platform [14,15].

### Mosquitoes

The mosquitoes used in these experiments were laboratory-reared *Anopheles arabiensis* (Ifakara strain, originally sourced from Sagamaganga village, Kilombero district in 2008) from the IHI insectaries. The larvae were fed on Tetramin® fish food and maintained at temperatures of 28 ± 1°C. Pupae were placed in emergence bowls inside a 30 × 30 × 30 cubic cm netted cage in a separate room where temperatures were maintained at 27 ± 3°C and relative humidity at 70-90%. A 10% glucose solution was supplied in the cages for the emergent adults. The insectary was maintained at 12:12 (light: dark) photoperiod, from 0600 hrs to 1800 hours (light period) and 1800 hrs to 0600 hrs (dark period). The mosquitoes used in these experiments were three to eight day-old nulliparous females. The mosquitoes were starved from sugar solution for six hours.

### Volunteers

Male volunteers, aged between 18 and 40 years were educated on aims, benefits and risks of the study and recruited on written informed consent. The use of strictly male volunteers was to prevent potential risk of malaria infection to pregnant female volunteers. All volunteers were highly experienced in performing human-landing catches. During the SFS experiments, volunteers were screened daily for parasitaemia using RDTs and if found positive, excluded from participating any further in the experiments and treated with artemether-lumefantrine (ALU), first-line drug for treatment of malaria in Tanzania. During the field evaluation, in addition to daily screening, volunteers were provided with mefloquine prophylaxis. The volunteers were instructed not to use any fragranced soap or perfume, tobacco or alcohol 12 hours before the start and throughout the experiments.

### Repellents

The repellent tested was donated by SC Johnson & Sons Inc (Racine, WI, USA). Three treatments were tested: 1) a lotion-based formulation containing 15% DEET as the active ingredient, being the test product; 2) 15% DEET diluted in absolute ethanol, being the standard control, and 3) a placebo made of a similar lotion formulation as the test product, but lacking the active ingredient, being the negative control. Technicians were blinded to the repellent application.

### Repellent application

To establish the amount of repellent required for application in the SFS experiments, surface area of the lower limbs of three adult male volunteers was determined by first measuring the length from ankle to the knee and the circumference of the ankle and knee using a tape measure. The surface area was then calculated using the formula that expresses the lower limb surface as a trapezium or cylinder:

(1) $$\mathrm{Area}=0.5\left(c_{a}+c_{k}\right)D_{\mathrm{ka}}$$

where c_a_ is the circumference of the ankle in cm, c_k_ circumference of the knee, and D_ka_ is the distance between c_a_ and c_k_.

Three volunteers were initially asked to apply the repellent *ad libitum* (the amount they felt was safe to protect from mosquito bites) to their legs. While applying the repellent, the volunteers wore latex gloves to avoid absorption of repellents into their skin, which would otherwise reduce the net quantity of repellent applied. The product bottles were then weighed using a precision weighing balance (Ohaus Corp, Pine Brook, NJ, USA) after this initial application to determine the amount applied by each volunteer. The average amount of repellent per volunteer was then calculated from these results. The average amount applied per volunteer was determined to be 2 mg per volunteer-leg. The average surface area of a volunteer’s leg was 1,041 cm^2^. The amount of DEET applied was 0.002 mg/cm^2^ (2 mg/1,041 cm^2^). After amount of repellent required for application was determined, the PI (SO) premeasured these amounts in a Petri dish for each volunteer every evening. The volunteers were then asked to wear latex gloves and apply their respective amounts on their lower limbs every evening before the start of each experiment.

### Study design

The SFS experiments used a partially randomized, 3 × 3 unbalanced Latin square design. The three treatments used in these experiments were assigned numbers: 1 (15% DEET lotion), 2 (15% DEET ethanol) and, 3 (placebo lotion). Three volunteers were used in these experiments and were randomly assigned to each of the three treatments using the lottery method. The volunteers were also randomly assigned sitting positions inside the SFS using the lottery method, and moved between the positions in the same order every night. One round of repellent evaluation was made up of three nights of mosquito collections, with each volunteer wearing a different treatment and sitting at a different position on each of these nights. A single set of three volunteers conducted these experiments for six nights (two rounds of repellent evaluation). For logistical reasons, the second set of three volunteers conducted the experiments for three nights (one round of repellent evaluation). Therefore, the mosquitoes were collected for a total of nine nights in the SFS, but with two different sets of volunteers. Data from the three rounds was pooled. The authors are aware that this limitation may have increased data variance because of individual variability in attraction of mosquitoes and efficiency in mosquito collections.

The PI (SO) premeasured the amounts of treatments 15 min prior (17.45), to the start of the experiments and asked the volunteers to apply their respective amounts on their lower limbs while wearing latex gloves. The volunteers had also been asked to put on knee-length shorts and ankle high boots, so as to standardize the area of exposure. The volunteers sat on low stools 10 m equidistant from each other in a triangular formation. A cage holding 100 mosquitoes was placed at the centre of this triangle formation. It was determined from literature that the biting rate in the study area was 62.5 bites/person/night [18]. Therefore, 100 mosquitoes were released in each hour in the SFS containing three volunteers to simulate the high biting pressure of the field setting. It was assumed that only half the number of all mosquitoes released would bite the volunteers. Therefore, each volunteer would have received approximately 67 bites/person/night. The average landing rates/volunteer/hour was also determined. At the top of every hour (18.00 h-22.00) the mosquitoes were released by one of the volunteers. The experiments were conducted from 18.00 because this was the reported time of the start of biting activity of vectors in the study area [19]. In total, four cages containing 100 mosquitoes each were used during each night of the SFS experiment. Each volunteer was given a head torch, which they switched on only when they felt a mosquito landing on their limb or when scanning the legs every 30 seconds for mosquitoes [20]. The volunteers were also given four paper cups, marked from the first to the fourth hour, and instructed to place the catches for each hour in their respective cups. The paper cups were covered with netting that had a hole at the centre to place the mosquitoes into the paper cups, which were plugged using a cotton wool to prevent mosquitoes from escaping. At the end of the experiment (22.00), the mosquitoes collected in the four paper cups were stored in the freezer at the IHI laboratory until the next morning. At 09.00 the next day, the mosquitoes in each paper cup were counted and recorded for each hour. The mosquitoes were then discarded and the paper cups cleaned ready for the day’s experiment.

### Field evaluation of topically applied repellents

Field evaluation of repellents was conducted in Mbingu village, described above. The experiments were conducted next to the rice fields and away from human dwellings to avoid potential bias in the number and behaviour of mosquitoes [21].

The field evaluation of repellents was conducted using a partially randomized, 3 × 3 balanced Latin square design, in the same manner as the SFS repellent evaluation described above. All field experiments were conducted at the site identified and described above. Six volunteers, two of whom also performed the SFS evaluations, were recruited for field evaluation of repellents. A first set of three volunteers conducted the repellent evaluation for nine nights, followed by the second set of volunteers who also conducted the experiment for nine nights at the same site. Therefore six volunteers evaluated the repellents for a total of 18 nights in the field as it was hypothesised that there would be greater variability in field data and more replicates would be required. The volunteers sat 20 m equidistant from each other in a triangular formation. They collected mosquitoes from 18.00 to 22.00, and placed them in the different paper cups marked one to four hours. At the end of the collections, the paper cups holding the mosquitoes were placed in a cool box containing a piece of cotton wool impregnated with chloroform, which killed the mosquitoes. The next morning the mosquitoes in each paper cup were counted by the respective volunteer and the numbers recorded. The mosquitoes were sorted into anophelines and culicines and stored in separate Petri dishes that were layered with cotton wool and silica gel to prevent desiccation. The mosquitoes were brought back to the IHI laboratory where the culicines were identified to species level by an experienced entomologist using taxonomic keys [22]. The *Anopheles gambiae* complex was identified to species level using polymerase chain reaction (PCR) [23].

### Statistical analysis

#### Calculation of percentage protection

Data from the SFS and field trials were recorded in a Microsoft Excel spreadsheet (Microsoft Corporation), with columns for the date, name of volunteer, treatment the volunteer was wearing, position the volunteer was sitting and the number of mosquitoes caught during each hour. This data was then exported into STATA 11 (StataCorp LP, College Station, Texas, USA), where the total number of mosquitoes caught when using 15% DEET lotion and 15% DEET in ethanol were compared to the total number of mosquitoes caught when using the placebo lotion for each night regardless of who was using it, and an average was calculated. The reductions in number of mosquitoes in these two treatments (15% DEET lotion and 15% DEET in ethanol) were designated protection and expressed as a percentage, (percentage protection). The formula used to calculate percentage protection is shown below:

(2) $$P=\left[C‒T\right]/C\times 100$$

where C is the number of mosquitoes caught when the volunteer was using the placebo lotion and T is number of mosquitoes caught when the volunteer was using either the 15% DEET lotion or 15% DEET ethanol.

These results for each night of collection were then aggregated and the average percentage protection when using either 15% DEET ethanol or 15% DEET ethanol calculated using STATA 11.

#### Poisson regression analysis

Count data was then fitted into a Poisson model in STATA 11, with a log link function and a random intercept for each row of data to account for over dispersion, so as to determine relative risk of being bitten by a mosquito. A Poisson model was chosen because it is used to model count data over a specified period of time, i.e. the number of mosquito bites occurring in one hour. It is also used to model rare events (mosquito bites), which is what was expected when a volunteer was wearing either 15% DEET lotion or 15% DEET ethanol. A Poisson model also allowed for analysis of repeated measures over time on the same individual, i.e. the number of mosquitoes caught by each individual on each day while wearing a different repellent and sitting at a different position. The number of mosquitoes caught/hour was fitted as the dependent variable, and interaction of repellent with time, individual variability and position fitted as predictors. Day (which also accounted for confounders like temperature, humidity and wind speed), was fitted as a random covariate, and a random intercept, in this case a Unique ID, was fitted into the model to account for over dispersion of the data.

The percentage protection of 15% DEET lotion and 15% DEET ethanol per hour and regression coefficients relative to the placebo (Incidence Rate Ratio, IRR) were determined to assess the decay of repellents through time.

### Ethical considerations

The volunteers used in these experiments were recruited on written informed consent. In case of any positive blood slide for malaria parasites, ALU combination therapy, the first-line drug for malaria treatment in Tanzania, was available. The volunteers were also informed of the study objectives and that they were free to withdraw their participation at any time during the experiments. The volunteers were experienced in human landing catch techniques and were issued with loose net jackets to prevent the mosquitoes biting the upper parts of the body. For field experiments, the volunteers were provided with mefloquine prophylaxis to protect them against contracting malaria. Ethical approval was granted by the Ethical Review Boards of Ifakara Health Institute (IHRDC IRB A46), the Tanzanian National Institute of Medical Research (NIMR/HQ/R8a/VOL IX/780), and London School of Hygiene of Tropical Medicine (LSHTM 5174).

## Results

### Semi-field experiments

#### Average percentage protection

The average percentage protection of 15% DEET lotion in the SFS as calculated from Equation 2 above was 82.13% (95% CI 75.93-88.82) and 71.29% (95% CI 61.77-82.28) for 15% DEET in ethanol over four hours of mosquito collection.

### Poisson regression analysis

The relative risk of being bitten by a mosquito over the four hour test when using 15% DEET lotion compared to placebo lotion was reduced by 91.8% (95% CI 85.73-95.79%, IRR = 0.082 z = −8.23, P <0.0001). When 15% DEET ethanol was compared to the placebo lotion, the relative risk of being bitten by mosquitoes was also reduced by 92.30% (95% CI 85.06-95.45%, IRR = 0.077, z = −8.21, P <0.0001) (Table 1). The relative risk of being bitten increased in hours two and three relative to hour one, although these differences were not significant. There was, however, a significant increase in the risk of being bitten in hour four compared to hour one for both 15% DEET lotion IRR = 3.71 (95% CI 1.78-7.78, z = 3.47, P = 0.001) and 15% DEET ethanol IRR = 3.43 (95% CI 1.60-7.39, z = 3.17, P = 0.002). This is an indication of repellent decay over time. There was location bias, with position 3 having a higher risk of being bitten compared to location one, IRR 2.00 (95% CI 1.51-2.66, z = 4.79, P <0.0001). Position 3 within the SFS was located closest to a nearby restaurant and the mosquitoes were probably more attracted to the light and human cues. There was variability in individual attractiveness to mosquitoes, (Table 1).

**Table 1:** **Effect of 15% DEET repellent over time, treatment, position and person on *Anopheles arabiensis *** **in a four-hour repellent evaluation in the semi-field system at Ifakara Health Institute**

**Treatments**	**Hours**	**Incidence rate ratio (IRR)**^**1 **^**[95% CI]**	**Z-test statistic**^**2**^	**P-value**^**3**^
15% DEET in ethanol	1	-	-	-
	2	1.744 [0.796-3.819]	1.39	0.164
	3	1.223 [0.559-2.675]	0.51	0.613
	4	3.708 [1.767-7.780]	3.47	0.001
15% formulated DEET repellent	1	-	-	-
	2	0.877 [0.359-2.140]	−0.29	0.774
	3	1.674 [0.756-3.709]	1.27	0.204
	4	3.439 [1.601-7.386]	3.17	0.002
**Treatments**				
Placebo	-	-	-	-
15% DEET in ethanol	-	0.082 [0.045-0.149]	−8.23	<0.0001
15% DEET in lotion format	-	0.077 [0.042-0.142]	−8.21	<0.0001
**Position**				
1	-	-	-	-
2	-	0.818 [0.587-1.139]	−1.19	0.236
3	-	2.000 [1.506-2.656]	4.79	<0.0001
**Person**				
1	-	-	-	-
2	-	0.619 [0.441-0.868]	−2.78	0.005
3	-	2.372 [1.796-3.133]	6.08	<0.0001

^1^The data for position one, person one and effect of treatments in hour one were used as a reference values for calculating the incidence rate ratios (IRR) for mosquito bites. ^2^The test statistic z is the ratio of the Coefficient to the Standard error of that respective predictor and is used to test against a two-sided alternative hypothesis that the Coefficient is not equal to zero. ^3^The probability (P) that a particular z test statistic is different to what has been observed under the null hypothesis.

### Field trial experiments

#### Mosquito species composition in the study area

**Figure 1:**
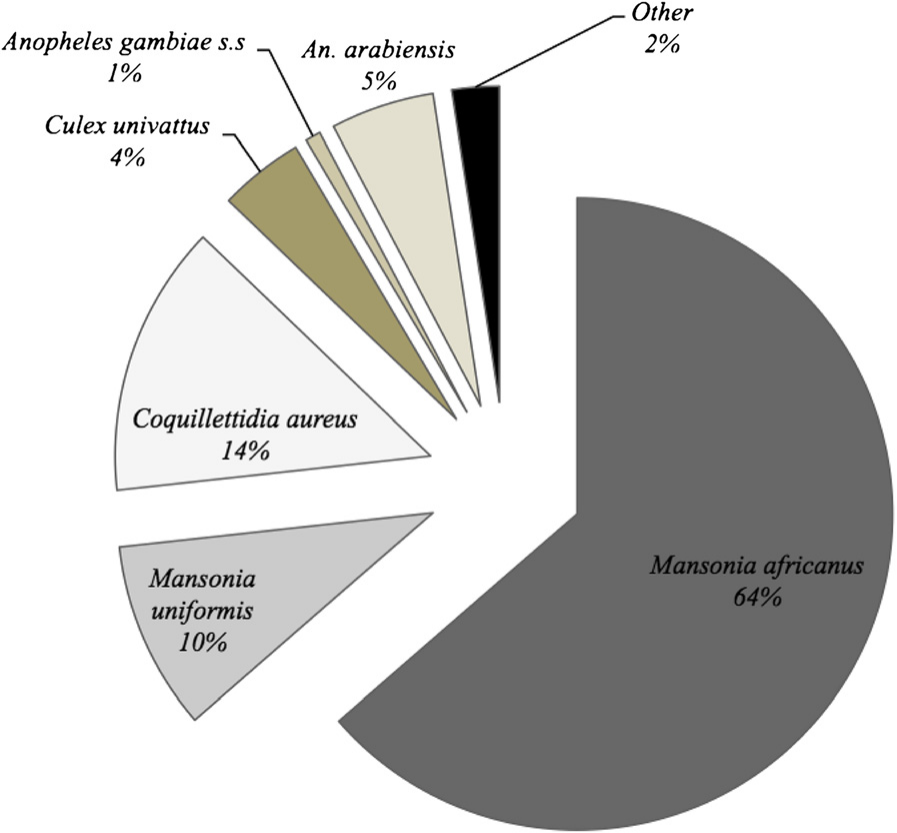
Pie chart showing mosquito species composition caught in Mbingu village during human landing catches sampled over 18 nights in field experiments at Mbingu village.

A total of 4,844 mosquitoes were caught in 72 hours over 18 nights. The catch included: 295 (5.4%) *An. gambiae s.l*. ,3,082 (64.6%) *Mansonia africanus*, 467 (9.8%) *Mansonia uniformis*, 673 (14.1%) *Coquillettidia aureus*, 210 (4.4%) *Culex univattus* and 177 (3.7%) other *Culex* species (Figure 1).

#### Anopheles gambiae s.l. composition in the study area

All the *An. gambiae s.l.* caught were identified to species level by PCR. Out of the 295 successful PCR amplifications, 12.88% (n = 38) were *An. gambiae s.s,* while 87.12% (n = 257) were *An. arabiensis* (Figure 1).

### Average percentage protection

The average percentage protection, of 15% DEET lotion in the field was 85.10% (95% CI 78.97-91.70) and 88.24% (95% CI 84.45-92.20) for DEET ethanol over four hours of mosquito collection, as calculated from Equation 2.

### Poisson regression analysis

The relative risk of being bitten by a mosquito over the four hour test when using 15% DEET lotion was reduced by 94.78% (95% CI 91.46-96.81%, IRR = 0.052, z = −11.74, P <0.0001) compared to the placebo lotion and 96.41% (95% CI 93.94-97.88%, IRR = 0.035, z = −12.42, P <0.0001) while using 15% DEET in ethanol (Table 2).

**Table 2:** Effect of 15% DEET repellent over time, treatment, position, and person on total number of mosquitoes in a four-hour repellent evaluation in the Mbingu village

**Treatments**	**Hours**	**Incidence rate ratio**^**1 **^**[95% CI]**	**Z-test statistic**^**2**^	**P-value**^**3**^
15% DEET in lotion format	1	-	-	-
	2	0.839 [0.422-1.667]	−0.50	0.618
	3	1.133 [0.578-2.222]	0.37	0.714
	4	1.699 [0.873-3.307]	1.56	0.118
15% DEET in ethanol	1	-	-	-
	2	0.791 [0.381-1.641]	−0.63	0.529
	3	2.049 [1.027-4.090]	2.04	0.042
	4	3.027 [1.524-6.011]	3.17	0.002
**Treatments**				
Placebo	-	-	-	-
15% DEET in lotion format	-	0.052 [0.038-0.085]	−11.74	<0.0001
15% DEET in ethanol	-	0.035 [0.021-0.060]	−12.42	<0.0001
**Position**				
1	-	-	-	-
2	-	1.091 [0.851-1.400]	0.69	0.498
3	-	0.876 [0.684-1.123]	−1.04	0.299
**Person**				
1	-	-	-	-
2	-	4.892 [3.511-6.816]	9.38	0.000
3	-	1.392 [0.973-1.987]	1.81	0.070
4	-	1.065 [0.624-1.820]	0.23	0.815
5	-	0.933 [0.54 0–1.611]	−0.25	0.804
6	-	1.377 [0.808-2.347]	1.18	0.239

^1^The data for position one, person one and effect of treatments in hour one were used as a reference values for calculating the incidence rate ratios (IRR) for mosquito bites. ^2^The test statistic z is the ratio of the Coefficient to the Standard error of that respective predictor and is used to test against a two-sided alternative hypothesis that the Coefficient is not equal to zero. ^3^The probability (P) that a particular z test statistic is different to what has been observed under the null hypothesis.

The risk of being bitten in the fourth hour increased three-fold compared to the first hour when using 15% DEET in ethanol IRR = 3.03 (95% CI 1.52-6.01, z = 3.17, P = 0.001). There was, however, no significant increase in the risk of bitten through hours 1 to 4 when using 15% DEET lotion repellent (Table 2). There was lower variability in individual attractiveness to mosquitoes, with only volunteer 2 being significantly more attractive to mosquitoes, IRR = 4.89 (95% CI 3.51-6.82, z = 9.38, P <0.0001). This individual was consistently more attractive in all field experiments. In this field study, the volunteers recruited had differing body mass. There were volunteers who had a larger body mass than this individual but caught fewer mosquitoes when they were compared. Also, even though all team members were highly experienced, there were more experienced field technicians who did not catch as many mosquitoes as this individual. All volunteers use the same concentration and gram/cm^2^ repellents per body surface area, ruling out the potential bias of one volunteer applying more repellent. Studies have shown variable responses of mosquitoes to singular or constituent host attractive cues. It is therefore likely that, the combination of this volunteers body cues/odours [24], made him more attractive to mosquitoes than the combination of cues that were emitted by the other volunteers.

### Anopheles gambiae experiments

Data on *An. gambiae s.l.* from the study area was analysed separately to determine the efficacy of repellents on this species of major medical importance.

### Average percentage protection

The average percentage protection of 15% DEET lotion in the field was 93.40% (95% CI 89.21-97.79) and 91.45% (95% CI 85.79-97.47) for 15% DEET in ethanol over four hours of mosquito collection, as calculated from Equation 2.

### Poisson regression analysis

The relative risk of being bitten when using 15% DEET lotion was reduced by 82.86% (95% CI 53.26-93.71, IRR = 0.171, z = −3.45, P = 0.001) when compared to placebo lotion and by 83.43% (95% CI 55.81-93.79, IRR = 0.165, z = −3.59, P <0.0001) when using15% DEET in ethanol over the four hours of the test. There was no significant difference in the average number of *An. gambiae s.l.* caught at the different positions in the field, in each hour or by each treatment in each hour over the four hours of mosquito collections demonstrating consistent protection. There was however a significant difference in the average number of *An. gambiae s.l.* caught by volunteer 2: IRR = 2.66 (95% CI 1.42-4.98, z = 3.06, P = 0.002) and volunteer 6: IRR 0.26 (95% CI 0.81-0.84, z = −2.25, P = 0.025) relative to volunteer 1 (Table 3).

**Table 3:** **Effect of 15% DEET repellent over time, treatment, position, and person on *Anopheles arabiensis *** **in a four-hour repellent evaluation in the Mbingu village**

**Treatments**	**Hours**	**Incidence rate ratio (IRR)**^**1 **^**[95% CI]**	**Z-test statistic**^**2**^	**P-value**^**3**^
15% DEET in lotion format	1	-	-	-
	2	0.403 [0.083-1.956]	−1.13	0.260
	3	0.326 [0.068-1.550]	−1.41	0.159
	4	0.722 [0.185-2.812]	−0.47	0.639
15% DEET in ethanol	1	-	-	-
	2	1.229 [0.343-4.399]	0.32	0.750
	3	1.963 [0.583-6.621]	1.09	0.277
	4	1.370 [0.400-4.693]	0.86	0.500
**Treatments**				
Placebo	-	-	-	-
15% DEET in lotion format	-	0.171 [0.063-0.467]	−3.45	0.001
15% DEET in ethanol	-	0.165 [0.062-0.441]	−3.59	<0.0001
**Position**				
1	-	-	-	-
2	-	0.932 [0.542-1.602]	−0.25	0.800
3	-	1.262 [0.750-2.126]	0.88	0.380
**Person**				
1	-	-	-	-
2	-	2.660 [1.420-4.979]	3.06	0.002
3	-	1.801 [0.924-3.510]	1.73	0.084
4	-	0.381 [0.127-1.141]	−1.72	0.085
5	-	0.328 [0.106-1.015]	−1.93	0.053
6	-	0.262 [0.081-0.841]	−2.25	0.025

^1^The data for position one, person one and effect of treatments in hour one were used as a reference values for calculating the incidence rate ratios (IRR) for mosquito bites. ^2^The test statistic z is the ratio of the Coefficient to the Standard error of that respective predictor and is used to test against a two-sided alternative hypothesis that the Coefficient is not equal to zero. ^3^The probability (P) that a particular z test statistic is different to what has been observed under the null hypothesis.

### Comparison of full field and semi-field system data

**Figure 2:**
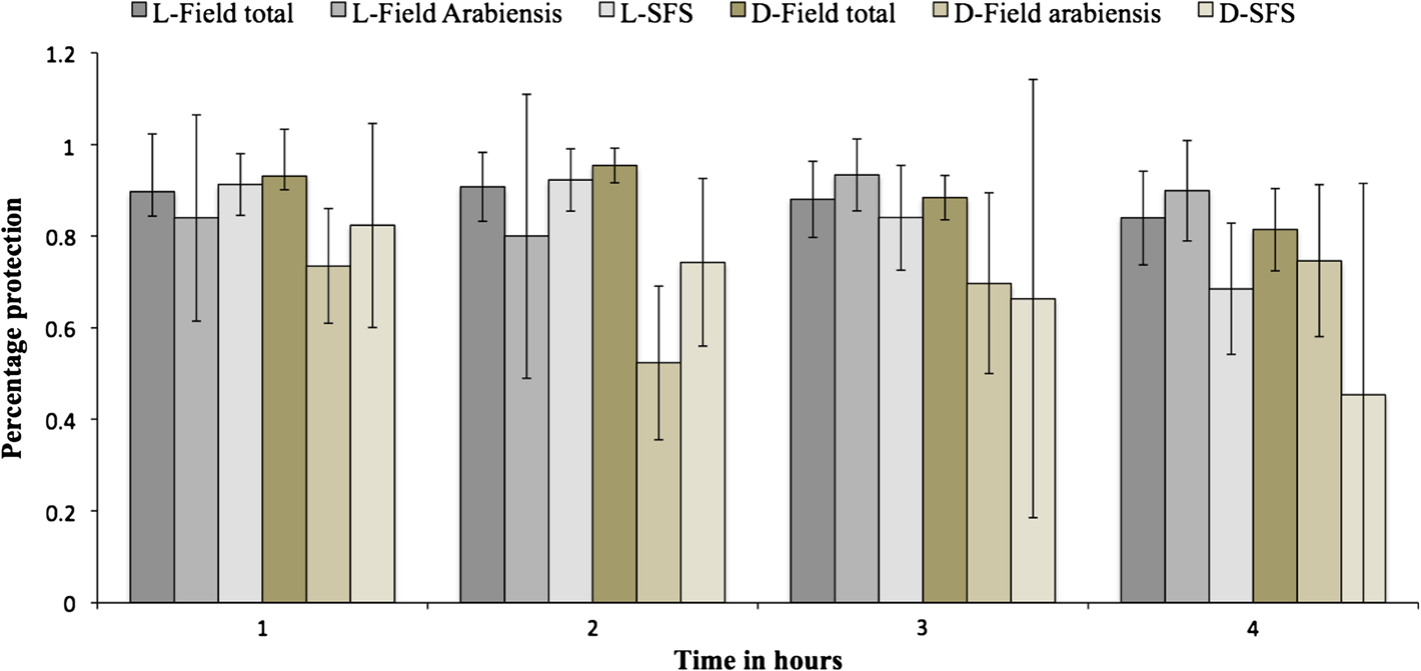
**Comparison of percentage protection of 15% DEET lotion repellent and 15% DEET ethanol against *Anopheles arabiensis *in the semi-field system, all mosquito species in the field and *Anopheles arabiensis *in the field after four hours of mosquito collection.** L-Field total is 15% DEET lotion tested against all mosquito species in the field. L-Field *Arabiensis* is 15% DEET lotion against *An. arabiensis* in the field. L-SFS is 15% DEET lotion against *An. arabiensis* in the semi-field system. D-Field total is 15% DEET in ethanol tested against all mosquito species in the field. D-Field *Arabiensis* is 15% DEET in ethanol against *An. arabiensis* in the field. D-SFS is 15% DEET in ethanol against *An. arabiensis* semi-field system.

Decay of repellent from the Poisson regression equations (Tables 1 and 2) and the linear regression demonstrated that 15% DEET in lotion format decayed at a slower rate than 15% DEET in ethanol in both the SFS and field settings. A linear regression also demonstrated a similar trend with regression coefficients showing a more rapid decay of 15% DEET in ethanol in the SFS and against all mosquitoes in the field, with equal decay of the two formulations against *An. gambiae s.l.* in the field (Table 4). However, the results from the linear regression equations (regression coefficients) should be interpreted with caution as the data were over dispersed even after transformation to a proportion (percentage protection) and Linear regression is a parametric test that assumes equal variance around the mean. The percentage protection provided by 15% DEET lotion and 15% DEET in ethanol was similar in the SFS and field settings and on both occasions both treatments provided greater protection in the field than in the SFS (Figure 2). When the two treatments (15% DEET lotion and 15% DEET ethanol) were compared statistically there was no difference between the two measured in the SFS IRR = 0.904 (95% CI 0.44-2.80, p *=* 0.833) nor the field IRR = 0.621 (95% CI 0.316-1.221, p = 0.168).

**Table 4:** **Comparison of rate of decay of repellents, percentage protection and log-transformed means of mosquito catches per hour in the semi-field system against *Anopheles arabiensis *** **and in the field against all mosquito species and *Anopheles arabiensis***

**Experiment**	**Hour**	**Regression equation**	**Treatments**	**GEOMEAN**	Percentage protection (CI)*
Semi-field evaluation against *An. arabiensis*	1	Y = −0.0765 + 1.0315	Lotion-based 15% DEET repellent	2.69	90.88 (84.25-98.03)
	2			1.7	91.85 (84.85-99.43)
	3			3.1	82.60 (70.39-96.93)
	4	R^2^ = 0.29138		4.63	65.97 (52.28-83.24)
	1	Y = −0.119x = 0.9685	15% DEET in ethanol	4.65	75.55 (51.79-110.20)
	2			3.63	70.76 (54.63-91.65)
	3	R^2^ = 0.08181		3.17	82.18 (61.19-110.36)
	4			6.26	58.42 (40.45-84.36)
Field evaluation against all mosquito species	1	Y = −0.0077x + 0.8921	Lotion-based 15% DEET repellent	4.77	87.39 (76.49-99.83)
	2			4.03	88.92 (79.15-99.88)
	3			5.44	85.99 (76.30-96.90)
	4	R^2^ = 0.00174		8.03	83.98 (73.78-94.19)
	1	Y = −0.0427x + 1.0009	15% DEET in ethanol	4.22	91.98 (84.14-100.55)
	2			5.94	95.11 (91.02-99.37)
	3	R^2^ = 0.11871		10.89	87.87 (83.08-92.95)
	4			13.5	79.03 (69.14-90.33)
Person					
Field evaluation against *An. arabiensis*	1	Y = 0.0311x + 0.7904	Lotion-based 15% DEET repellent	1.22	92.58 (83.18-103.05)
	2			1.25	100.00 (100.00-100.00)
	3			1	92.60 (84.30-101.72)
		0.06763			
	4			1.64	88.02 (76.15-101.75)
	1	Y = 0.0208 + 0.6235	15% DEET in ethanol	0.72	95.20 (87.33-103.78)
	2			0.94	94.93 (87.85-102.57)
	3	R^2^ = 0.045263		1.5	82.26 (61.18-110.61)
	4			1.17	91.15 (83.82-101.31)

*Some confidence intervals exceed 100% because the ranges were calculated by regression analysis using continuous data. They should therefore be read as 100% efficacy.

## Discussion

The epidemiology of malaria in sub-Saharan Africa is experiencing a subtle shift. Before the advent of LLINs and indoor residual spraying (IRS), malaria transmission was mediated indoors and late in the night mainly by *An. gambiae s.s*. This species of the *An. gambiae* complex is known to be predominantly anthropophilic, endophagic and endophilic [25,26]. This characteristic is responsible for the success of LLINs and IRS in controlling *An. gambiae s.s*., as these tools predominantly target indoor biting and resting malaria vectors. However, *An. arabiensis*, the other dominant vector species of the *An. gambiae* complex [26] exhibits a more plastic behaviour [27]. In areas where the host is predominantly human and found indoors, this vector displays anthropophilic, endopahgic and endophilic behaviour, similar to its sibling species, *An. gambiae s.s.* However in areas where the host are found outdoors and are non-human, *An. arabiensis* readily shifts to exophagic, exophilic and zoophagic behaviour [25]. Therefore, extensive and long-term employment of LLINs and IRS is likely to significantly diminish and in some situations completely eliminate the populations of *An. gambiae s.s.,* thereby selecting for the highly adaptable *An. arabiensis* that predominantly bites early in the evening and outdoors [27]. As a result, even though LLINs and IRS will decrease malaria transmission as a whole, there will be a substantial proportion of residual transmission occurring outdoors and in the early evenings that these intradomiciliary tools cannot tackle [27].

Consequently, there is a need to develop novel tools or methods that can tackle this residual transmission. Repellents, both topical and spatial, provide a promising solution for controlling outdoor transmission [28-30]. However before topical repellents are employed in the community, their performance needs to be correctly and accurately measured under user conditions. It is, therefore, essential to develop a robust methodology for testing repellent efficacy that is representative of conditions under which the repellents are used (the community), but does not expose individuals conducting these experiments to potential malaria vectors [1,2]. It was hypothesized that locating the SFS in regions representative of ambient conditions for the targeted disease vector and testing repellents on humans against these vectors is likely to yield results that correlate well with field tests. Therefore, to qualify the effect of these treatments in these two settings, data for *An. arabiensis* in the SFS was analyzed against data of *An. gambiae s.l.* in the field experiments (as > 80% of this species complex was found to be *An. arabiensis*).

The findings demonstrated that 15% DEET lotion protected against 82.13% (95% CI 75.93-88.82) of the bites in the SFS compared to 93.40% (95% CI 89.21-97.79) protection against bites in the field, while 15% DEET in ethanol protected against 71.29% (95% CI 61.77-82.28) bites in the SFS compared 91.45% (95% CI 85.79-97.47) bites in the field against *An. gambiae s.l.* These results demonstrate that both 15% DEET lotion and 15% DEET repellent were more efficacious in the field than in the SFS. A plausible explanation for this might be the high biting pressure observed in the SFS compared to the field. Mosquitoes were exposed to fewer hosts than they normally would in the field and their numbers were continuously increased from 100 mosquitoes in the first hour to 400 mosquitoes in the fourth hour (Figure 2, Tables 5 and 6). By simulating high biting pressure that increased over time as is seen in the field due to the circadian rhythm of the local malaria vectors [19], the authors ensured that the repellent worked extremely well against the predominant malaria vector species before going to the more dangerous field setting. It is known that repellents have varying effects on the other mosquito species present in the field [6,31]. As a result, the effect of the repellent in the field might be over or underestimated depending on the other species present in the field. It is, therefore, prudent, that before the effect of a repellent is established, it should be tested against different mosquito species to assess its efficacy. These data showed that DEET efficacy against one Anopheline species only in the SFS was similar to that for a range of non-anophelines in the full field although this needs to be validated for other repellent classes, as not all repellents are broad-spectrum.

**Table 5:** **Mean landing rates (MLR) of *An. arabiensis*/volunteer/hour in a four hour repellent evaluation in the Semi-field system at the Ifakara Health Institute**

	**Volunteer 1 median (IQR)**	**Volunteer 2 median (IQR)**	**Volunteer 3 median (IQR)**
***Placebo***			
Hour 1	17 (6–20)	22 (11–27)	41 (19–46)
Hour 2	16 (13–19)	18 (8–18)	17 (16–43)
Hour 3	14 (10–24)	24 (6–29)	37 (18–56)
Hour 4	14 (11–30)	16 (8–20)	28 (12–36)
***15% DEET in ethanol***			
Hour 1	0	0	12 (1–13)
Hour 2	1 (0–3)	1 (0–5)	8 (7–10)
Hour 3	1 (0–1)	0 (0–1)	9 (6–19)
Hour 4	4 (1–10)	4 (0–4)	19 (7–18)
***15% DEET in lotion formulation***			
Hour 1	2 (0–4)	0 (0–1)	4 (2–6)
Hour 2	1 (1–5)	2 (0–2)	1 (1–5)
Hour 3	3 (2–15)	2 (0–2)	3 (2–4)
Hour 4	3 (2–17)	3 (2–5)	8 (4–10)

**Table 6:** **Mean landing rates of *An. gambiae *** **s.l/volunteer/hour in a four hour repellent evaluation in Mbingu village**

	**Volunteer 1 median (IQR)**	**Volunteer 2 median (IQR)**	**Volunteer 3 median (IQR)**	**Volunteer 4 median (IQR)**	**Volunteer 5 median (IQR)**	**Volunteer 6 median (IQR)**
***Placebo***						
Hour 1	10 (2–10)	2 (0–3)	4 (1–5)	0 (0–2)	0 (0–6)	(0)
Hour 2	2 (1–7)	4 (2–4)	3 (1–4)	2 (0–5)	1 (0–3)	1 (0–3)
Hour 3	4 (1–22)	3 (0–6)	10 (1–13)	0 (0–3)	0 (0–4)	0 (0–4)
Hour 4	4 (0–6)	3 (1–7)	11 (3–12)	0 (0–8)	2 (0–3)	0 (0–5)
***15% DEET in ethanol***						
Hour 1	0 (0–0)	2 (1–9)	0 (0–1)	0 (0–0)	0 (0–0)	0 (0–0)
Hour 2	0 (0–1)	1 (0–7)	2 (0–6)	0 (0–0)	0 (0–0)	0 (0–0)
Hour 3	0 (0–3)	4 (1–8)	2 (0–4)	0 (0–5)	0 (0–0)	0 (0–0)
Hour 4	0 (0–0)	4 (1–5)	3 (1–6)	0 (0–0)	0 (0–0)	0 (0–1)
***15% DEET in lotion***						
Hour 1	0 (0–0)	0 (0–1)	0 (0–1)	0 (0–1)	0 (0–2)	1 (0–1)
Hour 2	0 (0–0)	0 (0–2)	0 (0–0)	1 (0–1)	0 (0–0)	0 (0–0)
Hour 3	0 (0–1)	1 (0–1)	0 (0–0)	0 (0–0)	0 (0–1)	0 (0–0)
Hour 4	0 (0–0)	2 (0–3)	0 (0–2)	0 (0–0)	0 (0–0)	0 (0–0)

It is often assumed that formulated repellents provide longer protection against arthropod bites, especially those that have a high vapour pressure. However, findings from this study demonstrate that this may not always be true, and that different formulations of repellents containing the same amount of active ingredient (AI) provide relatively similar efficacy against arthropod bites. These findings are similar to a study carried out to test the efficacy of different formulations of repellents against ticks [32,33].

This is the first study known to have compared the efficacy of topical repellent in both the SFS and field and to determine a correlation between these two settings. However, the current study did suffer from some shortcomings, and an attempt to outline a rationale procedure for conducting future studies incorporating the lessons learnt from this study is suggested below.

A fully randomized, balanced Latin square design should be employed, so that each volunteer tests each of the repellents in all positions available in the SFS. Each volunteer should test each treatment for an equal number of days in each position. The treatments and positions should be randomly assigned to the volunteers and the movement through these positions should be also be randomized. The exact number volunteers testing the repellents should be established, and this number used to calculate the average repellent dose to be applied per individual/surface area. This is to avoid under or overestimating the repellent dose required per person in a case where fewer or more individuals are used to establish the amount of repellent required than those actually testing the repellents. Each group of volunteers testing the repellents should perform an equal number of replicates so that the results are not confounded by individual variability in attractiveness of mosquitoes, a bias that is minimised when all volunteers have equal number of replicates. All repellent application should be done by an individual wearing gloves, either by the volunteers themselves or an assistant, to prevent repellent absorption into the skin, thereby reducing net amount of repellent being applied. The local dominant vector species, the biting rate per night and time of biting should be established and the number of mosquitoes representative of the biting rate used in the study. The experiments should also be started at the beginning of peak biting activity of the dominant vector in the local area, to avoid interfering with the circadian rhythm. Varying the biting pressure and peak biting times may vary the results of the SFS.

Using a new model of repellent efficacy as a function of user compliance and malaria intensity developed by SJM and Briet (personnal communication), the predicted reduction in malaria provided by the repellent in this scenario would be 44%, assuming 80% repellent efficacy and 80% compliance among users with a sporozoite index of 0.005637 (Okumu, personnal communication), a transmission season of 200 days per year and biting pressure of 32 bites per night from the major malaria vector *An. arabiensis*[34]*.*

## Conclusion

The findings of this study support the hypothesis that repellent testing conducted in SFS yields similar results to field tests, and could be used in place of field tests, to avoid unnecessary exposure of volunteers to potentially infectious disease vectors, provided repellent efficacy is established against a range of representative mosquito species.

## Competing interests

The authors declare that they have no competing interests.

## Authors’ contributions

SJM and SO conceived the study; SO, EM, DL, HN, DL, JK, and EM performed data collection; MM identified mosquitoes; SO and SJM performed analysis; SO wrote the manuscript; SJM and MM commented on the manuscript. All authors have agreed to the final version.
